# Molecular analysis and prenatal diagnosis of seven Chinese families with genetic epilepsy

**DOI:** 10.3389/fnins.2023.1165601

**Published:** 2023-05-12

**Authors:** Bin Mao, Na Lin, Danhua Guo, Deqin He, Huili Xue, Lingji Chen, Qianqian He, Min Zhang, Meihuan Chen, Hailong Huang, Liangpu Xu

**Affiliations:** ^1^Medical Genetic Diagnosis and Therapy Center, Fujian Maternity and Child Health Hospital, College of Clinical Medicine for Obstetrics and Gynecology and Pediatrics, Fujian Medical University, Fuzhou, China; ^2^Fujian Provincial Key Laboratory of Prenatal Diagnosis and Birth Defect, Fuzhou, China

**Keywords:** genetic epilepsy, molecular analysis, pathogenic variants, prenatal diagnosis, *Alu* elements

## Abstract

**Introduction:**

Genetic epilepsy is a large group of clinically and genetically heterogeneous neurological disorders characterized by recurrent seizures, which have a clear association with genetic defects. In this study, we have recruited seven families from China with neurodevelopmental abnormalities in which epilepsy was a predominant manifestation, aiming to elucidate the underlying causes and make a precise diagnosis for the cases.

**Methods:**

Whole-exome sequencing (WES) combined with Sanger sequencing was used to identify the causative variants associated with the diseases in addition to essential imaging and biomedical examination.

**Results:**

A gross intragenic deletion detected in *MFSD8* was investigated via gap-polymerase chain reaction (PCR), real-time quantitative PCR (qPCR), and mRNA sequence analysis. We identified 11 variants in seven genes (*ALDH7A1, CDKL5, PCDH19, QARS1, POLG, GRIN2A*, and *MFSD8*) responsible for genetic epilepsy in the seven families, respectively. A total of six variants (c.1408T>G in *ALDH7A1*, c.1994_1997del in *CDKL5*, c.794G>A in *QARS1*, c.2453C>T in *GRIN2A*, and c.217dup and c.863+995_998+1480del in *MFSD8*) have not yet been reported to be associated with diseases and were all evaluated to be pathogenic or likely pathogenic according to the American College of Medical Genetics and Genomics (ACMG) guidelines.

**Methods:**

Based on the molecular findings, we have associated the intragenic deletion in *MFSD8* with the mutagenesis mechanism of *Alu*-mediated genomic rearrangements for the first time and provided genetic counseling, medical suggestions, and prenatal diagnosis for the families. In conclusion, molecular diagnosis is crucial to obtain improved medical outcomes and recurrence risk evaluation for genetic epilepsy.

## 1. Introduction

Epilepsy, characterized by recurrent seizures, is a heterogeneous group of conditions with different clinical presentations, drug responses, etiologies, and prognoses (EpiPM Consortium, 2015). As one of the most common neurological disorders, epilepsy has affected ~ 0.5–1% of the global population (Thurman et al., 2011; Beghi, 2020). Epilepsy can be roughly subdivided into two forms: common epilepsy, which accounts for ~ 95% of the total affected cases and manifests variable symptoms and complicated etiologies, and the rare form, some cases of which could exhibit typical Mendelian inheritance (Thakran et al., 2020). The International League against Epilepsy (ILAE) used to categorize epilepsy into idiopathic generalized and focal conditions, depending on whether a single or both cerebral hemispheres are involved (Guerri et al., 2020); nevertheless, untypical seizures like infantile spasms belong to neither of them (Berg et al., 2010). As per the latest ILAE guideline released in 2017, etiology has been utilized as the classification criteria: genetic, structural, infectious, metabolic, immune, and unknown epilepsy (Thakran et al., 2020).

Genetic epilepsy constitutes a proportion of all human epilepsy where genetic defects are the only or dominant factor leading to seizures and other neuropsychiatric comorbidities (Willmore and Ueda, 2002; Pandolfo, 2013). Copy number variations (CNVs) in specific loci of the chromosomes have been confirmed as susceptible to epilepsy (Striano and Minassian, 2020). For example, researchers have associated chromosomes 8q13–q21 (FEB1), 19p (FEB2), 2q23–q24 (FEB3), 5q14–q15 (FEB4), 6q22–q24 (FEB5), and 18p11 (FEB6) with febrile seizures that are induced by high fever (Audenaert et al., 2006). In addition, an increasing number of genes have been identified as responsible for genetic epilepsy with definitive Mendelian inheritance (George, 2004).

The inclusion standard of genes related to genetic epilepsy has been controversial due to the heterogeneous phenotypes of the corresponding neurological syndromes and developmental encephalopathies (Dunn et al., 2018). Guerri et al. (2020) summarized that at least 96 genes had been associated with genetic disorders characterized by epilepsy. The functions of these epilepsy-related genes are various, including but not limited to neuronal excitability, synaptic transmission, network development, and especially neuronal metabolism (Pandolfo, 2013). The interference of neuronal metabolism generally results from inborn errors of metabolism (IEM) and presents as biochemical pathway dysfunction and finally, epileptic seizures (Vitiello et al., 2012). There was an estimation that ~ 42% of monogenic disorders with epilepsy belong to IEM (Tumiene et al., 2018). The pathophysiology of metabolic epilepsy could be a disturbance in substrates, products, by-products, energy utilization, etc. (Reddy and Saini, 2021).

The majority of genetic epilepsy manifests as neurological syndromes or developmental encephalopathies with multiple neurodevelopmental abnormalities (Walsh and Mccandless, 2001). The common comorbidities of monogenic disorders characterized by epileptic seizures consist of intellectual/psychomotor development delay, myoclonus, autism spectrum disorders, electroencephalogram (EEG) abnormalities, hypotonia, microcephaly, and characteristic faces. Apart from the overlapping phenotypes among different genetic disorders with epileptic seizures, variable expressivity and incomplete penetrance are also common in these conditions. Although most genetic disorders characterized by genetic seizures face the dilemma of negative response to anti-epileptic drugs, irreversible neurological symptoms, or unsatisfying clinical outcomes, precision treatment to certain monogenic epilepsy syndromes, especially those caused by IEM, has been proven to be effective (Sharma and Prasad, 2017). Consequently, elucidating the molecular basis of epilepsy involved with monogenic defects is expected to facilitate the precise diagnosis, pathogenesis, and prognosis and, finally, satisfying treatment concerning the diseases.

In this study, we recruited seven unrelated families from China with unidentified syndromes characterized by epileptic seizures. We collected the clinical information, including ultrasound, computed tomography (CT), magnetic resonance imaging (MRI), and EEG examination, if available, and performed high throughput sequencing to detect the potential genetic causes of the conditions and make a precise diagnosis accordingly.

## 2. Materials and methods

### 2.1. Subjects

We recruited seven families suspected of genetic epilepsy with candidate variants from China since November 2021 (G003, G029, G046, G053, G060, G064, and G065), including three male and five female patients (two of the affected individuals G064-1 and G064-3 were daughter and father) and excluding those with negative genetic findings. All probands and their parents signed the written informed consent (the signature of participants under the age of 18 years was acquired from their parents) and were recruited in this project. Available clinical information of the affected individuals and peripheral blood samples of the subjects were obtained. This study was approved by the Institutional Review Board (IRB) of the Fujian Maternity and Child Health Hospital (2023KYLLR01016).

### 2.2. WES

All probands (and their parents) underwent WES to detect candidate variants responsible for the phenotypes. Genomic DNA was extracted from peripheral blood and purified on the KingFisher Flex System (Thermo Fisher Scientific, Waltham, MA); it was then treated with ultrasonic fragmentation, followed by purification through 60 M Agilent SureSelect Human All Exon V6 kit (Agilent, Santa Clara, CA). Paired-end sequencing was subsequently performed for captured DNA fragments via NovaSeq 6000 Sequencing System (Illumina, San Diego, CA).

Raw data were processed with the general bioinformatics analytic pipelines. The human GRCh37/hg19 genome was used as the reference sequence. Clean reads were trimmed from the raw data with the removal of adapters using the tool Trimmomatic (version 0.39) (Bolger et al., 2014) and were aligned to the reference genome using the Burrows-Wheeler Aligner (BWA) software package (Li and Durbin, 2009). The variant discovery was performed using the single nucleotide polymorphism (SNP) calling software Genome Analysis Toolkit (GATK, version 4.2.1.0; Broad Institute, Cambridge, MA). NxClinical (version 5.0; BioDiscovery, Hawthorne, CA) was run in parallel to detect single nucleotide variants (SNVs), CNVs, and loss of heterozygosity (LOH).

Variants were annotated through ANNOVAR and InterVar (http://wintervar.wglab.org/) sequentially (Wang et al., 2010; Li and Wang, 2017). Databases for annotation involving population frequency are as follows: Exome Aggregation Consortium (ExAC; http://exac.broadinstitute.org/), Genome Aggregation Database (gnomAD; http://gnomad.broadinstitute.org/), 1,000 Genomes Project (http://www.1000genomes.org/), and the database of SNP (dbSNP; http://www.ncbi.nlm.nih.gov/snp/); bioinformatics prediction tools involved are as follows: Polymorphism Phenotyping v2 (PolyPhen-2, version 2.2.3; http://genetics.bwh.harvard.edu/pph2/), Scale-Invariant Feature Transform (SIFT, version 1.1.5; http://sift.jcvi.org/), Mutation Taster 2021 (https://www.genecascade.org/MutationTaster2021/), Combined Annotation Dependent Depletion (CADD, version 1.6; http://cadd.gs.washington.edu/), and the integrated database VarCards (http://varcards.biols.ac.cn/) (Kumar et al., 2009; Adzhubei et al., 2010; Kircher et al., 2014; Li et al., 2018; Steinhaus et al., 2021); and phenotypes or disorders are as follows: Online Mendelian Inheritance in Man (OMIM; https://www.omim.org/), ClinVar (http://www.ncbi.nlm.nih.gov/clinvar/), and Human Phenotype Ontology (HPO; http://hpo.jax.org/app/). The pathogenicity of variants was classified as pathogenic (P), likely pathogenic (LP), variants of uncertain significance (VUS), likely benign (LB), and benign (B) in conformity with the American College of Medical Genetics and Genomics (ACMG) guidelines. After annotation of the variants, candidate variants supposed to be responsible for the diseases were selected based on the symptoms of patients, inheritance mode, genotype-phenotype correlations, and pathogenicity of the variants.

### 2.3. CNV-seq

Six probands (G003-1, G029-1, G046-1, G053-1, G064-1, and G065-1) were also subjected to CNV-seq in order to detect any pathogenic chromosomal imbalance. Genomic DNA was first broken into random fragments, followed by library construction via NEBNext Ultra II DNA Library Prep Kit for Illumina (New England Biolabs, Ipswich, MA) in accordance with the manufacturer's instructions. Paired-end DNA fragments were also sequenced on NovaSeq 6000 Sequencing System (Illumina) with an average production of read amount over 60 M (3 ×). Similar to WES, raw data from CNV-seq were processed with Trimmomatic and BWA to obtain clean reads and align to the reference human genome, followed by CNV calling via NxClinical. Numerous databases were used for the annotation and interpretation of CNVs, including gnomAD, OMIM, ClinVar, HPO, ClinGen (http://www.clinicalgenome.org/), and Database of Genomic Variation and Phenotype in Humans using Ensembl Resources (DECIPHER; http://www.deciphergenomics.org/).

### 2.4. Sanger sequencing

Sanger sequencing was conducted to verify the prospective pathogenic variants detected in the probands through WES and perform co-segregation analysis in the family. Reference sequences (hg19) of the genes *ALDH7A1* (NM_001182.5), *CDKL5* (NM_003159.3), *PCDH19* (NM_001184880.2), *QARS1* (NM_005051.3), *POLG* (NM_002693.3), *GRIN2A* (NM_000833.5), and *MFSD8* (NM_152778.4 and NC_000004.11) were acquired from the University of California, Santa Cruz (UCSC) Genome Browser database (http://genome.ucsc.edu/). Primers were designed using the software Primer Premier 5 (version 5.00; PREMIER Biosoft, Palo Alto, CA) as appropriate (listed in Supplementary Table S1). Targeted regions containing the candidate variants were amplified using polymerase chain reaction (PCR) with *LA Taq* DNA Polymerase with GC Buffer (TaKaRa, Shiga, Japan) and were then sequenced using the Applied Biosystems 3500xl DNA Analyzer (Thermo Fisher Scientific, Waltham, MA). Sequencing results of the amplicons were aligned to reference sequences via the CodonCode Aligner (version 6.0.2; CodonCode, Centerville, MA).

### 2.5. Real-time quantitative PCR

Real-time qPCR was performed to detect the deletion of exon 10 (c.863+995_998+1480del) in *MFSD8* for family G065. Primers targeted for the deletion region and internal reference sequence in chromosome 21 were listed in Supplementary Table S1. The reaction was run with TB Green *Premix Ex Taq* II (Tli RNaseH Plus; TaKaRa) on the Applied Biosystems StepOnePlus Real-Time PCR System (Thermo Fisher Scientific), following the manufacturer's instructions. At least three technical replicates for each sample were required.

### 2.6. RNA analysis

To investigate how the deletion of exon 10 in *MFSD8* affects the splicing pattern and mRNA expression, fresh peripheral blood of G065-1 to G065-3 was treated with RNAiso Blood (TaKaRa) and extracted as per standard procedures. After concentration measurement through NanoDrop 2000 Spectrophotometers (Thermo Fisher Scientific), 2 μg RNA of each sample was treated with the removal of genomic DNA and reverse transcription (RT) into cDNA using the PrimeScript RT reagent Kit with gDNA Eraser (TaKaRa). Targeted cDNA sequences of the three individuals were amplified and subjected to T-clone sequencing (primers listed in Supplementary Table S1).

### 2.7. Prenatal diagnosis

Based on the molecular findings, we provided prenatal gene diagnosis for five families where variants supposed to be responsible for the genetic epilepsy were identified (G003, G029, G053, G060, and G065). Amniocentesis was performed to collect a reasonable amount of amniotic fluid for molecular testing of the fetuses. Amniotic fluid cells both before (directly from centrifuge of the original amniotic fluid) and after cell culture were, respectively, extracted to obtain genomic DNA using the QIAamp DNA Mini Kit (QIAGEN, Hilden, Germany). Ultrasound and MRI examinations were taken as assisted evidence for disorders where the affected fetuses might have imaging phenotypes, if appropriate.

## 3. Results

### 3.1. Clinical manifestations

#### 3.1.1. Overview

**Figure 1:**
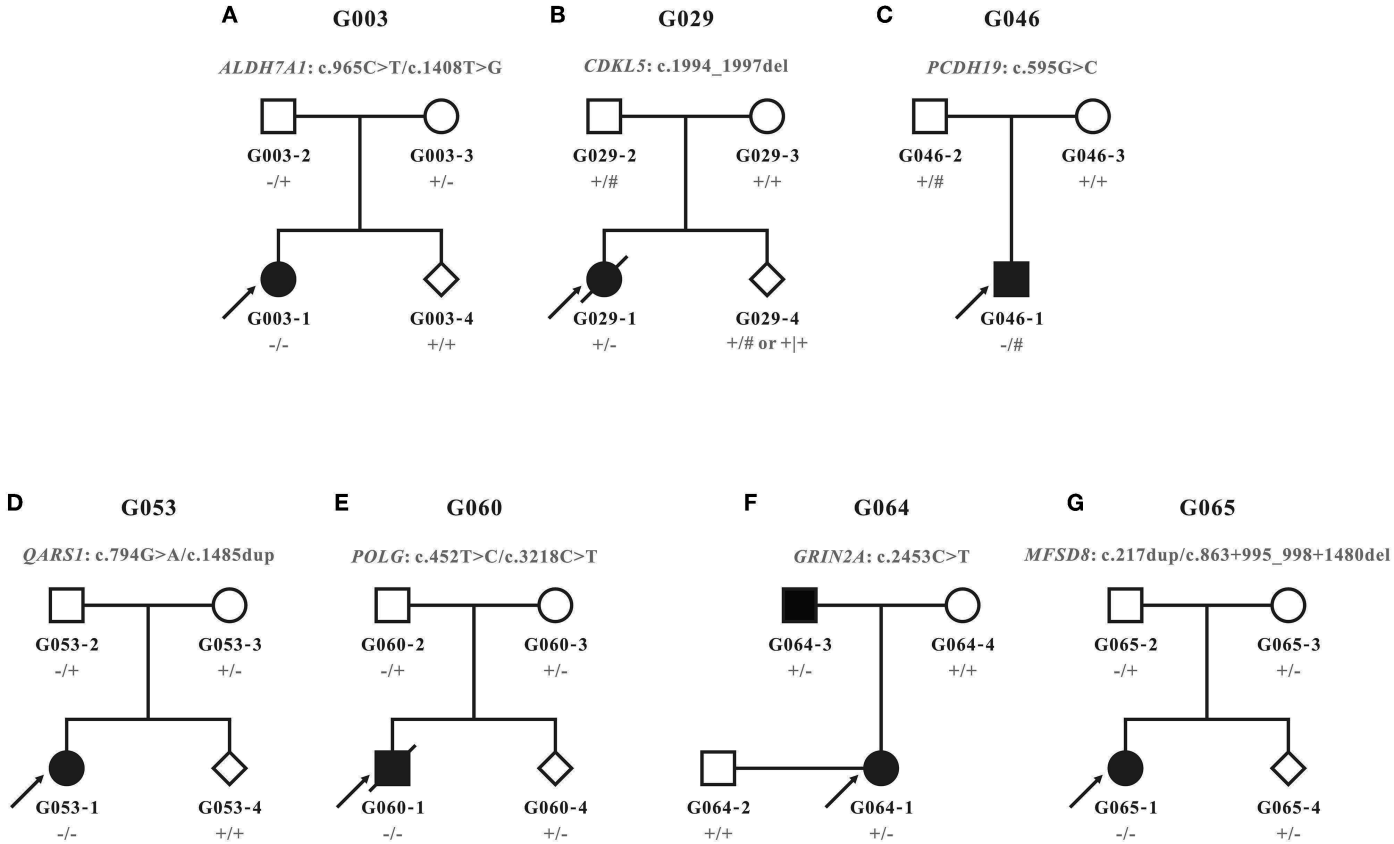
Pedigrees recruited in this study. **(A–G)** Pedigrees and genotypes of families G003, G029, G046, G053, G060, G064, and G065. Wild types and carriers of the corresponding variants were denoted with the plus (+) and minus signs (−), respectively. Male hemizygotes with only one allele in chromosome X were marked with the pound sign (#).

Eight patients with neurological syndromes characterized by epilepsy from seven unrelated families were included in this study (Figure 1). Among them, only one pedigree had a positive family history (G064). As shown in Table 1, all patients exhibited various degrees of intellectual disability and other neurological symptoms in addition to epileptic seizures, and two probands died of complications of the diseases (G029-1 and G060-1). Various abnormalities were found in the MRI (4/5) and EEG (3/5) examination of most patients. Other common symptoms included small for gestational age (2/8) and patent foramen ovale (2/8).

**Table 1:** Clinical manifestations of patients recruited in this study.

**Patient**	**Gender**	**Age at last visit**	**Family history**	**Epilepsy**	**Developmental delay**	**MRI**	**CT**	**EEG**	**MS/MS**	**GC-MS**	**Neuropsychic examination**	**Remarks**
G003-1	F	4 yr	N	Y	Global, moderate	Abnormal signal intensity, morphological changes	N/A	Abnormal	N/A	N/A	N/A	Microcephaly
G029-1	F	3 mo	N	Y	Global, severe	Physiological hydrocephalus	N/A	Abnormal	Normal	Normal	N/A	Anemia, died after last visit
G046-1	M	4 yr	N	Y	Intellectual, mild	Normal	N/A	Normal	N/A	N/A	N/A	Ocular hypertelorism, high arched palate
G053-1	F	2 yr	N	Y	IGR; global, moderate	Macrogyria	Leukoaraiosis	N/A	N/A	N/A	N/A	Maternal diabetes, SGA, metabolic acidosis, PFO, hypertonia
G060-1	M	4 mo	N	Y	Global, severe	N/A	Normal	Sporadic sharp wave, spike-and-wave complexes	Normal	Increased LAC, PA, 3-HBA, 3-HIBA, 2,4-HPLA	HINE: 38	SGA, myocardial damage, hypoammonemia, hyperlactatemia, hepatic insufficiency, PFO, tricuspid regurgitation; died at 6 months old
G064-1	F	27 yr	Y	Y	Intellectual, moderate	N/A	N/A	N/A	N/A	N/A	WAIS-RC FSIQ: 18	Autism spectrum disoder
G064-3	M	49 yr		Y	Intellectual, moderate	N/A	N/A	N/A	N/A	N/A	N/A	Father of G064-1
G065-1	F	4 yr	N	Y	DR; global, moderate	Extracerebral space expanded	N/A	Normal	N/A	N/A	Mean GDS DQ: 34.1, ASLS: 18, CARS: 35	Gait instability, slight hypertonia of right knee, visual deterioration

2,4-HPLA, 2,4-hydroxyphenyllactic acid; 3-HBA, 3-hydroxybutyrate; 3-HIBA, 3-hydroxyisobutyric acid; ASLS, Infant-Junior Middle School Student's Ability of Social Life Scale; CARS, Childhood Autism Rating Scale; CT, computed tomography; DQ, developmental quotient; DR, developmental regression; EEG, electroencephalogram; F, female; FSIQ, full scale intelligence quotient; GC-MS, gas chromatography-mass spectrometry; GDS, Gesell Developmental Schedules; HINE, Hammersmith Infant Neurological Examination; IGR, intrauterine growth retardation; LAC, lactic acid; M, male; mo, month; MRI, magnetic resonance imaging; MS/MS, tandem mass spectrometry; N, no; N/A, not available; PA, pyruvic acid; PFO, patent foramen ovale; SGA, small for gestational age; WAIS-RC, Wechsler Adult Intelligence Scale-Revised by China; Y, yes; yr, year.

#### 3.1.2. MRI

G003-1: Symmetrically distributed abnormal signal intensity in the brain white matter of bilateral ventricles and subcortical areas of bilateral cerebral hemispheres indicated a high possibility of metabolic encephalopathy. Furthermore, the morphological changes of bilateral frontal, temporal, and parietal lobes suggested the occurrence of neuronal migration disorders (macrogyria).

G029-1: Mild external physiological hydrocephalus was observed (extracerebral space slightly expanded).

G046-1: No obvious abnormality was found.

G053-1: Extensive edema changes were seen in the brain white matter of bilateral cerebral hemispheres. The cerebral sulcus of bilateral cerebral hemispheres was shallow and sparse, and the surface of the brain was smooth, indicating the possibility of macrogyria.

G065-1: Partial extracerebral space slightly expanded, which indicated the possibility of decreased cerebral parenchymal volume.

Other patients (G060-1, G064-1, and G064-3) did not undergo MRI, or their examination results were unavailable.

#### 3.1.3. CT

G053-1: Decreased density of the brain white matter (leukoaraiosis) was observed in bilateral frontal, temporal, and parietal lobes, which indicated the possibility of congenital atelencephalia.

G060-1: No abnormality was found in intracranial regions.

Other patients (G003-1, G029-1, G046-1, G064-1, G064-3, and G065-1) did not undergo CT, or their examination results were unavailable.

#### 3.1.4. EEG

G003-1 and G029-1 showed abnormal EEG, while no obvious abnormality was found in G046-1 or G065-1.

G060-1: No obvious abnormality of background rhythms and epileptiform discharge was found in awake EEG. Sporadic sharp waves and spike-and-wave complexes were occasionally seen in the right parietal-occipital brain regions.

Other patients (G053-1, G064-1, and G064-3) did not undergo CT, or their examination results were unavailable.

#### 3.1.5. Biomedical analysis

G029-1: No obvious abnormality for the IEM examination of blood tandem mass spectrometry (MS/MS) or urine gas chromatography-mass spectrometry (GC-MS) was found.

G060-1: The patient was negative for blood MS/MS but showed increased lactic acid, pyruvic acid, 3-hydroxybutyrate, 3-hydroxyisobutyric acid (suggesting ketonuria), and 2,4-hydroxyphenyllactic acid (probably secondary to hepatic function damage) for urine GC-MS.

Other patients (G003-1, G046-1, G053-1, G064-1, G064-3, and G065-1) did not undergo biomedical analysis, or their analysis results were unavailable.

#### 3.1.6. Neuropsychic examination

G060-1: A total score of 38 (functions of the cranial nerves: 7, body posture: 3, movement: 6, tone: 18, and reflexes and reactions: 4) for the Hammersmith Infant Neurological Examination (HINE), an early examination tool for infants between 2 and 24 months old suspected of cerebral palsy, was evaluated at the age of 3 months and 16 days (for infants between 2 and 4 months of age, a HINE score under 57 is predicted to be cerebral palsy, with a score under 40 almost cerebral palsy).

G064-1: The score for the Wechsler Adult Intelligence Scale-Revised by China (WAIS-RC) was 27, 24, and 18, respectively, for verbal intelligence quotient (VIQ), performance IQ (PIQ), and full-scale IQ (FSIQ), which all fell at the 0.10th percentile.

G065-1: The developmental quotient (DQ) of Gesell Developmental Schedules (GDS) was 44.0 (16.5 months), 32.0 (12.0 months), 41.0 (15.5 months), 24.0 (9.0 months), 30.4 (11.5 months), and 34.1 (12.9 months) for gross motor, fine motor, adaptability, language, social skill, and general development (average) at the age of 37.8 months, which was classified as severe intellectual disability. The Infant-Junior Middle School Student's Ability of Social Life Scale was 18 (standard score: 8) and classified as mild intellectual disability. The total score of the Childhood Autism Rating Scale (CARS) was 35 and was evaluated as no child autism.

Other patients (G003-1, G029-1, G046-1, G053-1, and G064-3) did not undergo neuropsychic examination, or their examination results were unavailable.

### 3.2. CNV-seq

No P/LP CNVs were detected in any of the six probands (G003-1, G029-1, G046-1, G053-1, G064-1, and G065-1).

### 3.3. Gross deletion in *MFSD8*

**Figure 2:**
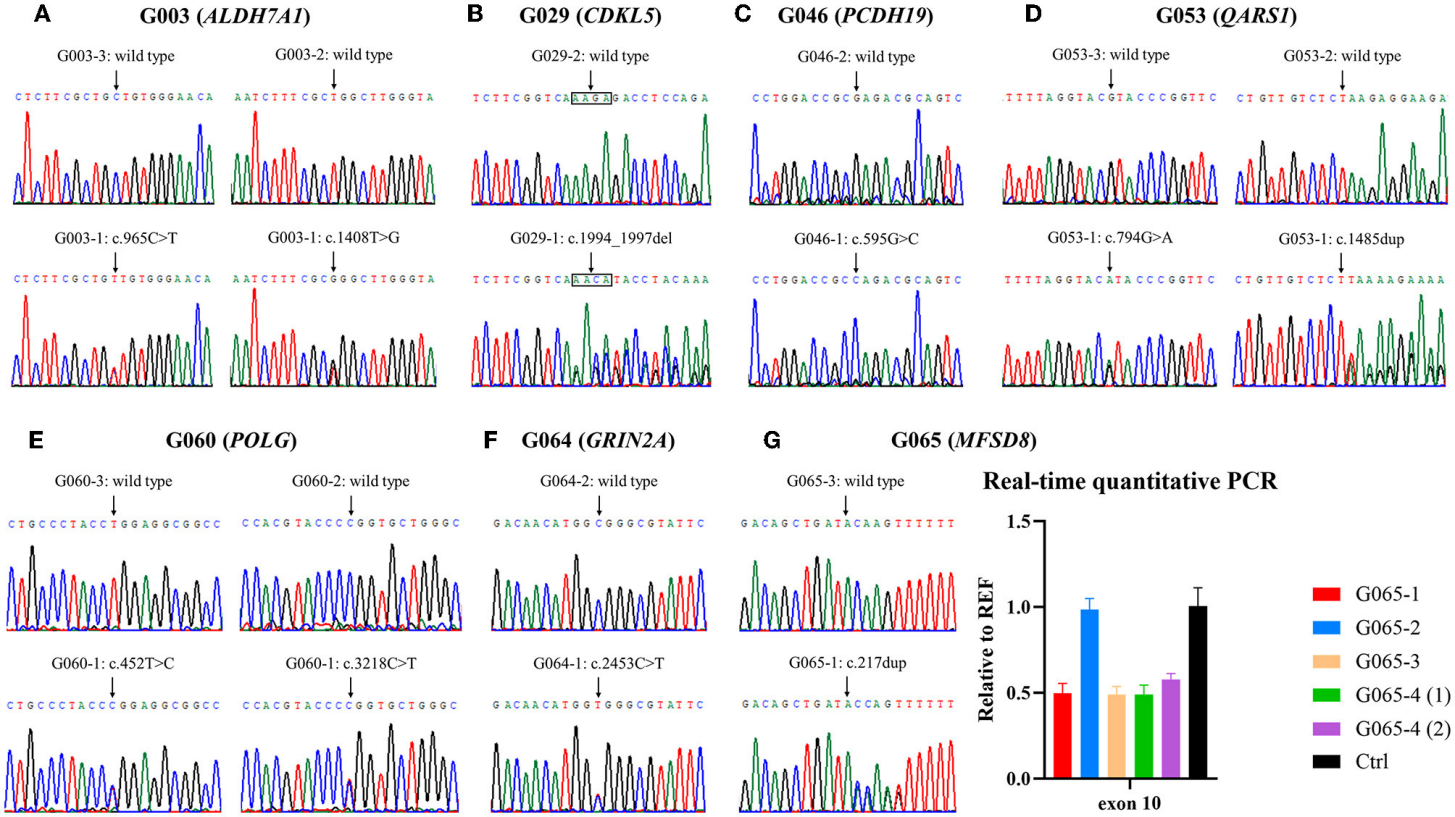
Validation of variants identified by whole-exome sequencing (WES). **(A–F)** Electropherograms of the variants detected in families G003, G029, G046, G053, G060, and G064. **(G)** Small variant and gross deletion in family G065 were confirmed using Sanger sequencing and real-time quantitative polymerase chain reaction (qPCR), respectively. The numbers (1) and (2) separately represent the samples extracted from amniotic fluid cells of the fetus G065-4 before and after cell culture. Ctrl, control.

**Figure 3:**
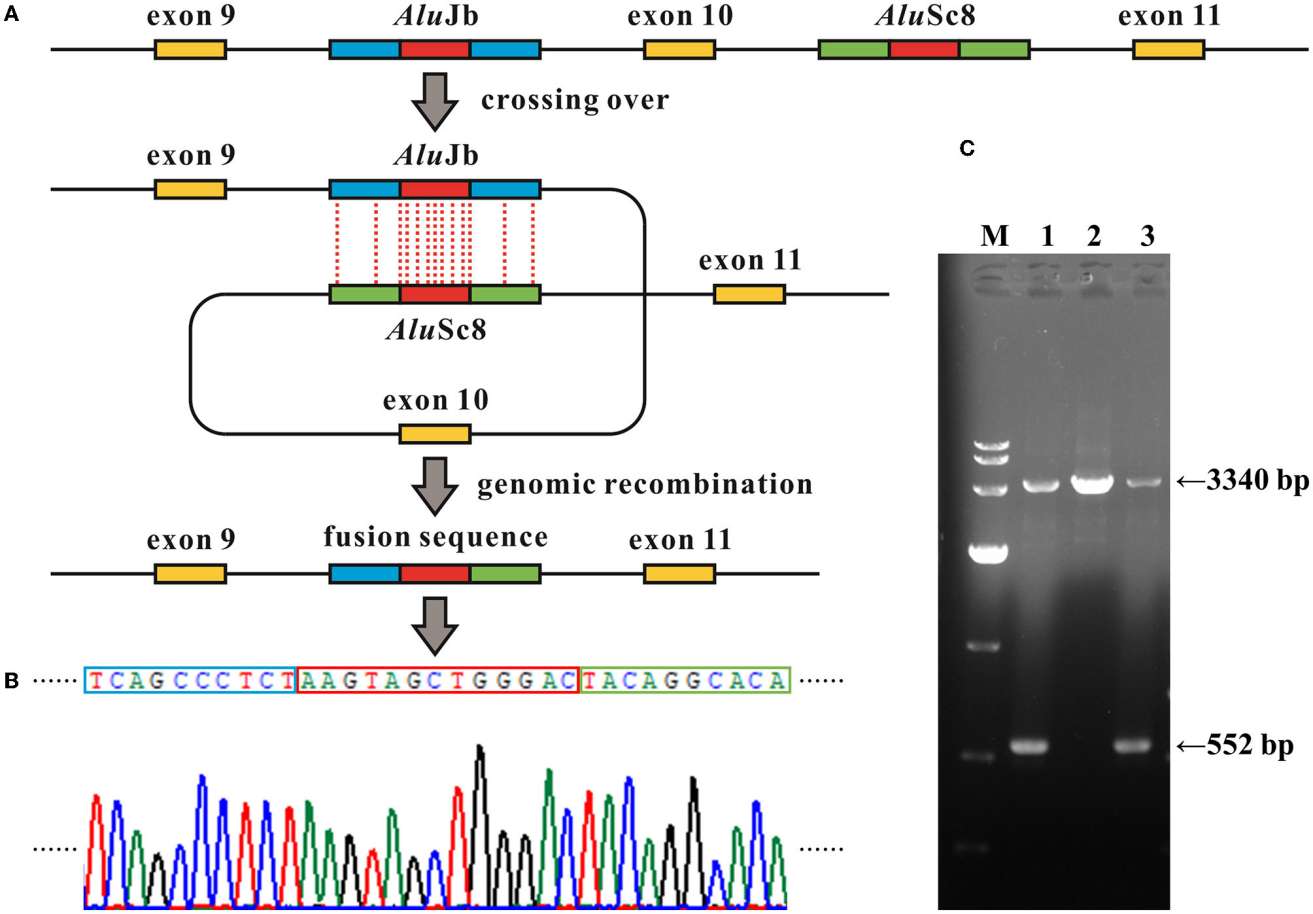
*Alu* recombination-mediated intragenic deletion identified in *MFSD8*. **(A)** Schematic diagram of the genomic deletion involving loss of exon 10 in *MFSD8* triggered by the recombination between two homologous *Alu* elements with a 13-bp identical core sequence (the red blocks). **(B)** Electropherograms of the sequences around the breakpoints of the deletion. The red box denotes the common fusion sequence as marked in **(A)**. **(C)** Gap-PCR suggests a gross deletion in family G065. Primer pairs of *MFSD8*-Gap-F and *MFSD8*-Gap-R (Supplementary Table S1) were used to amplify the fragments covering the 2,788-bp deletion. The results indicate that the proband G065-1 and her mother G065-3 were heterozygous carriers of intragenic deletion. M, DL 10,000 DNA Marker; 1, G065-1; 2, G065-2; 3, G065-3.

**Figure 4:**
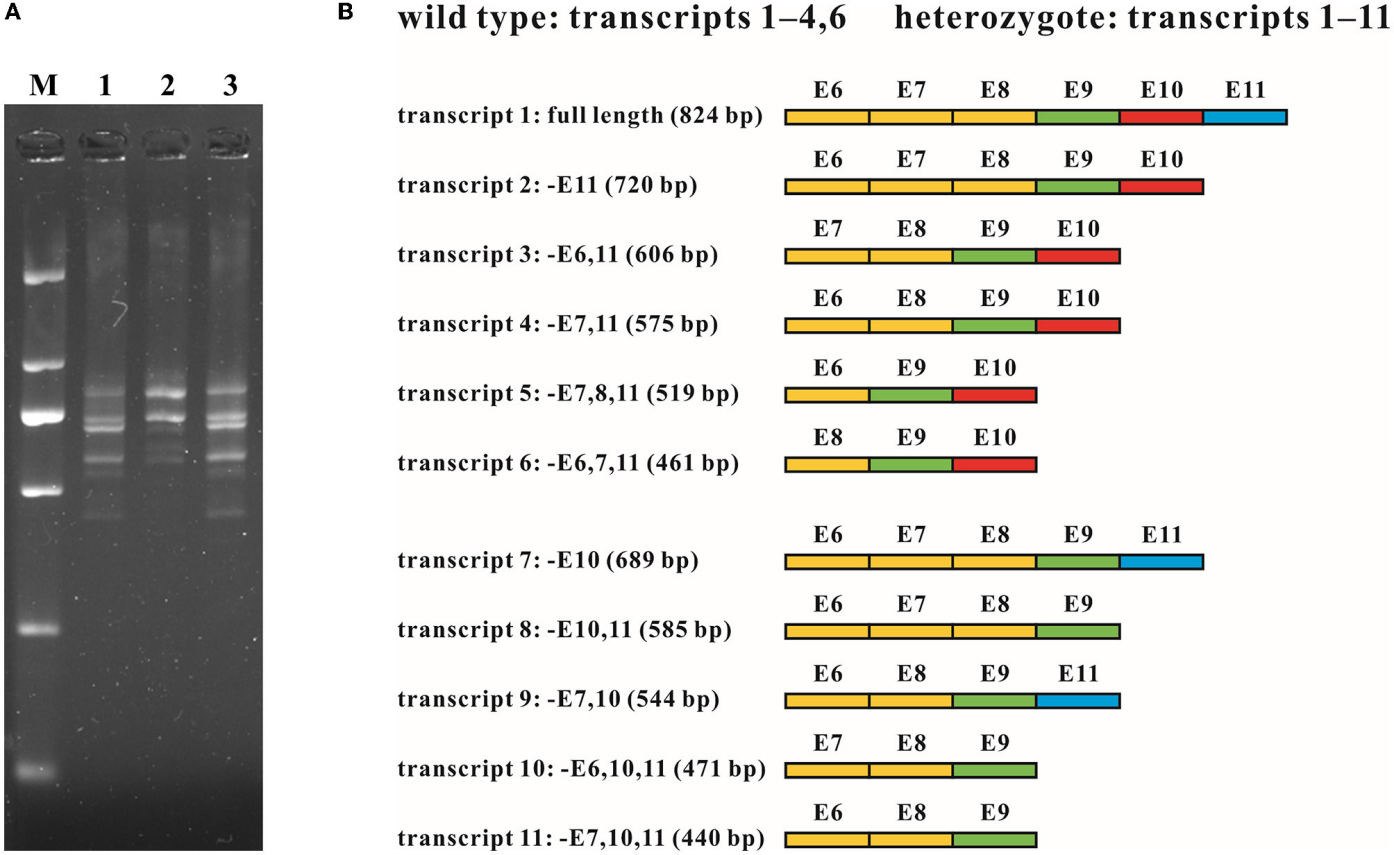
RNA analysis of the intragenic deletion covering exon 10 of *MFSD8*. **(A)** cDNA fragments targeting exons 6 to 11 of family G065. Multiple transcripts were observed in both the wild type (G065-2) and heterozygotes of the deletion (G065-1 and G065-3). M, DL 2,000 DNA Marker; 1, G065-1; 2, G065-2; 3, G065-3. **(B)** Schematic diagram of the sequence composition of transcripts detected in the wild type and heterozygotes of the deletion. Exons previously known to be involved in alternative splicing (exons 6–8) are marked in yellow, constitutive exons (exon 9) in green, exons within the deletion (exon 10) in red, and exons accidentally found to be subjected to alternative splicing (exon 11) in blue. E, exon; –, absence (of certain exons).

The WES analysis of family G065 suggested heterozygous loss of the tenth exon of *MFSD8* in G065-1 and her mother G065-3, while the copy number of adjacent exon 9 and exon 11 was normal. We performed real-time qPCR for validation of the deletion and prenatal diagnosis (Figure 2G). To determine the exact breakpoints, multiple pairs of primers were designed to amplify and sequence the fragment containing the deletion (Supplementary Table S1). The variant turned out to be a 2,788-bp deletion (c.863+995_998+1480del) including the whole of exon 10 and part of intron 9 and intron 10 (Figure 3). RNA analysis suggested that the gross deletion led to the skipping of exon 10. Given that exon 10 is present in all currently known transcripts of *MFSD8*, the gross deletion was considered to meet the PVS1 standard of the ACMG guidelines (see Section 3.5). Unexpectedly, the absence of exon 11 was also observed in various transcripts of individuals with and without gross deletion (Figure 4).

### 3.4. Candidate variants

According to the clinical characteristics and results of WES, 11 candidate variants in seven different genes were identified in each of the pedigrees (Table 2; Figure 2): c.965C>T/p.(Ala322Val) and c.1408T>G/p.(Trp470Gly) in *ALDH7A1* in family G003 with pyridoxine-dependent epilepsy (EPD; OMIM 266100), c.1994_1997del/p.(Lys665Argfs^*^118) in *CDKL5* in family G029 with developmental and epileptic encephalopathy 2 (DEE2; OMIM 300672), c.595G>C/p.(Glu199Gln) in *PCDH19* in family G046 with DEE9 (OMIM 300088), c.794G>A/p.(Arg265His) and c.1485dup/p.(Lys496^*^) in *QARS1* in family G053 with progressive microcephaly with seizures and cerebral and cerebellar atrophy (MSCCA; OMIM 615760), c.452T>C/p.(Leu151Pro) and c.3218C>T/p.(Pro1073Leu) in *POLG* in family G060 with mitochondrial DNA depletion syndrome 4A (MTDPS4A; OMIM 203700), c.2453C>T/p.(Ala818Val) in *GRIN2A* in family G064 with focal epilepsy with speech disorder and with or without impaired intellectual development (FESD; OMIM 245570), and c.217dup/p.(Thr73Asnfs^*^12) and c.863 + 995_998 + 1480del/g.128850371_128853158del in *MFSD8* in family G065 with neuronal ceroid lipofuscinosis 7 (CLN7; OMIM 610951). There were seven missense variants, three out-of-frame small deletions/duplications, and one gross deletion among the 11 variants. Co-segregation validation was carried out in all seven pedigrees (Figure 1). In family G064 with autosomal dominant (AD) inheritance, the variant was detected in the proband G064-1 and her affected father G064-3. For other pedigrees, the inheritance modes were either X-linked dominant (XLD) with *de novo* variants (2/7) or autosomal recessive (AR) from both carrier parents (4/7). The population frequency and bioinformatics prediction of the variants are listed in Supplementary Table S2.

**Table 2:** Variants associated with genetic epilepsy.

**Proband**	**Gene**	**Phenotype (OMIM#)**	**Reference sequence**	**Inheritance**	**Zygote type**	**Origin**	**Location**	**Nucleotide change**	**Amino acid change**	**Pathogenicity**	**Novelty**
G003-1	*ALDH7A1*	EPD (266100)	NM_001182.5	AR	C-het	F	E11	c.965C>T	p.(Ala322Val)	P: PS4 + PM2 + PM3 + PP3 + PP4	N
						M	E15	c.1408T>G	p.(Trp470Gly)	LP: PM2 + PM3 + PP3 + PP4	Y
G029-1	*CDKL5*	DEE2 (300672)	NM_003159.3	XLD	het	*de novo*	E13	c.1994_1997del	p.(Lys665Argfs*118)	P: PVS1 + PS2 + PM2	Y
G046-1	*PCDH19*	DEE9 (300088)	NM_001184880.2	XLD	hemi	*de novo*	E1	c.595G>C	p.(Glu199Gln)	LP: PS2 + PM2 + PP3	N
G053-1	*QARS1*	MSCCA (615760)	NM_005051.3	AR	C-het	F	E10	c.794G>A	p.(Arg265His)	LP: PM2 + PM3 + PP1 + PP4	Y
						M	E16	c.1485dup	p.(Lys496*)	P: PVS1 + PS4 + PM2 + PM3	N
G060-1	*POLG*	MTDPS4A (203700)	NM_002693.3	AR	C-het	F	E2	c.452T>C	p.(Leu151Pro)	LP: PM2 + PM3 + PP3 + PP4	N
						M	E20	c.3218C>T	p.(Pro1073Leu)	P: PS4 + PM2 + PM3 + PP3 + PP4	N
G064-1	*GRIN2A*	FESD (245570)	NM_000833.5	AD	het	F	E13	c.2453C>T	p.(Ala818Val)	LP: PM1 + PM2 + PM5 + PP1	Y
G065-1	*MFSD8*	CLN7 (610951)	NM_152778.4	AR	C-het	F	E5	c.217dup	p.(Thr73Asnfs*12)	P: PVS1 + PM2 + PM3	Y
						M	I9–I10	c.863+995_998+1480del	p.?	P: PVS1 + PM2 + PM3	Y

AD, autosomal dominant; AR, autosomal recessive; C-het, compound heterozygous; CLN7, neuronal ceroid lipofuscinosis 7; DEE, developmental and epileptic encephalopathy; E, exon; EPD, pyridoxine-dependent epilepsy; F, father; FESD, focal epilepsy with speech disorder and with or without impaired intellectual development; hemi, hemizygous; het, heterozygous; I, intron; LP, likely pathogenic; M, mother; MSCCA, progressive microcephaly with seizures and cerebral and cerebellar atrophy; MTDPS4A, mitochondrial DNA depletion syndrome 4A; N, no; OMIM, Online Mendelian Inheritance in Man; P, pathogenic; XLD, X-linked dominant; Y, yes.

### 3.5. Pathogenicity of the variants

Four variants identified have been associated with corresponding diseases as per previous research studies and/or archived as P/LP variants in the databases such as HGMD and ClinVar: c.965C>T in *ALDH7A1* (Ville et al., 2013; Yang et al., 2014), c.595G>C in *PCDH19* (Depienne et al., 2009), c.1485dup in *QARS1* (Zhang et al., 2014; Kodera et al., 2015), and c.3218C>T in *POLG* (Kurt et al., 2010; Baruffini et al., 2011). The variant c.452T>C in *POLG* has only been reported to be responsible for sensory ataxic neuropathy, dysarthria, and ophthalmoparesis (OMIM 607459) (Li et al., 2021), a disorder with some overlapping phenotypes with MTDPS4A from which the proband G060-1 suffered; this variant was then interpreted to be LP (PM2 + PM3 + PP3 + PP4) according to the ACMG guidelines. In addition, we have also identified six causative variants that have not been previously reported: c.1408T>G in *ALDH7A1* (LP: PM2 + PM3 + PP3 + PP4); c.1994_1997del in *CDKL5* (P: PVS1 + PS2 + PM2); c.794G>A in *QARS1* (LP: PM2 + PM3 + PP1 + PP4); c.2453C>T in *GRIN2A* (LP: PM1 + PM2 + PM5 + PP1) (Strehlow et al., 2019); and c.217dup (P: PVS1 + PM2 + PM3) and c.863+995_998+1480del (P: PVS1 + PM2 + PM3) in *MFSD8*. All six aforementioned novel variants have been submitted to ClinVar (accessions: SCV003853240, SCV003853245, and SCV003853278-SCV003853281).

### 3.6. Prenatal diagnosis

Prenatal diagnosis through amniocentesis was provided for five families (G003, G029, G053, G060, and G065). Family G029 had a strong desire for prenatal gene diagnosis, although we had informed them that the recurrence risk of a *de novo* variant is extremely low, except for the rare cases of genital gland chimera. The molecular diagnosis of the amniotic fluid cells confirmed that the fetus G029-4 was of wild type for the mutant site. For the other four families with a typical AR inheritance, two (G003-4 and G053-4) carried neither of the variants from parents, and the other two (G060-4 and G065-4) were carriers of the maternal variants (Figure 1).

## 4. Discussion

In this study, we identified the monogenic defects for each of the seven families with genetic epilepsy from China. In addition to the common symptom of epileptic seizures as a predominant phenotype, these individuals also exhibited various forms of developmental delay and neuropsychiatric manifestations including cerebral structural and EEG abnormalities. For example, the probands G003-1 and G053-1 were suggested to manifest macrogyria or pachygyria, which is defined as the increased size of cerebral gyri (often with a decreased number of cerebral sulci). Macrogyria/pachygyria falls on the wide spectrum of cortical developmental malformations, and its most severe form is known as lissencephaly (complete agyria), caused by variants in genes involving centrosome proteins (*PAFAH1B1*/*LIS1*), tubulins (*TUBA1A, TUBB, TUBB2A, TUBB2B, TUBB3, TUBB4A, TUBG1, TUBGCP2*, and *TUBGCP6*), microtubule motor proteins (*DYNC1H1*), actin-associated proteins (*DCX*), reelin (*RELN* and *VLDLR*), and forebrain development (*ARX*) (Mochida, 2009; Koenig et al., 2021; Kolbjer et al., 2021). Macrogyria or simplified gyri was a common imaging phenotype in patients with *QARS1* variants (Chan et al., 2022), and Johannesen et al. (2019) reported that 56% (10/18) of the *QARS1*-associated patients exhibited cortical structural anomalies including macrogyria. To the best of our knowledge, macrogyria has not been previously described in EPD although other malformations of cortical development were generally observed (Mercimek-Mahmutoglu et al., 2012; Jansen et al., 2014; Coci et al., 2017). Nevertheless, diffuse swelling of the cerebral gyri was reported in the autopsy findings of a patient with *ALDH7A1* causative variants (Marguet et al., 2016). We hypothesize that the cortical anomalies of macrogyria in G003-1 were secondary to seizures of recurrent intractable epilepsy. This resemblance in neurological characteristics suggested that multiple genes are involved in the neurodevelopmental process, and turbulences in these different pathways can result in overlapping phenotypes like epilepsy (Guerri et al., 2020). Based on the molecular findings of clinically indistinguishable epilepsy, the complex etiology of epileptic seizures and the significance of gene diagnosis of neurological disorders on suspicion of genetic epilepsy were highlighted (Lindy et al., 2018; Striano and Minassian, 2020).

Despite having numerous similar neuropsychiatric symptoms, the patients recruited in this study showed a wide range of severity in the phenotypic spectrum from mild intellectual disability to premature death in infancy. Furthermore, disease-specific phenotypes were observed in the corresponding affected individuals. For example, patient G003-1 with EPD presented a favorable response to pyridoxine supplementation, and the seizures stopped completely upon treatment as the causative gene *ALDH7A1* encodes the alpha-aminoadipic semialdehyde dehydrogenase involved in the catabolic pathways of lysine; characteristic liver failure was observed in proband G060-1, which was a common cause of death besides the status epilepticus for patients with MTDPS4A before the age of 3 years (Table 1). Thus, compared to common epilepsy that generally does not severely affect mental or cognitive functions, genetic epilepsy could be deemed a neurological syndrome with seizures as the predominant external manifestation (Thakran et al., 2020).

In family G065, WES analysis suggested a heterozygous loss of exon 10 of *MFSD8* in proband G065-1 and her mother G065-3, while the copy numbers of adjacent exons all remained normal. We then confirmed the deletion and provided prenatal diagnosis using samples from the amniotic fluid of fetus G065-4 using real-time qPCR (Figure 2G). Gap PCR primers that targeted the prospective deletion region (from the upstream of intron 9 to the downstream of intron 10) were designed in order to determine the exact breakpoint of the intragenic deletion overlapping exon 10. We tried multiple pairs of primers due to the homologous sequences in this region and amplification failure and managed to find two pairs of primers that could amplify both the wild-type and mutant alleles (Figure 3; Supplementary Figure S1). It turned out that the CNV was a 2,788-bp deletion from intron 9 to intron 10 and had a 13-bp sequence identical at both ends (5′-AAGTAGCTGGGAC-3′). The deletion was described as c.863+995_998+1480del in conformity with the 3′rule nomenclature of the Human Genome Variation Society (HGVS) (den Dunnen et al., 2016).

Further analysis revealed that the two common fusion sequences at both ends of the breakpoints were located within two *Alu* elements, *Alu*Jb (chr4:128852983–128853282) and *Alu*Sc8 (chr4:128850195–128850507), respectively. The similarity of the two repetitive sequences was 75%, which was calculated by the multiple sequence alignment tool Clustal X (version 2.1; Conway Institute, University College Dublin, Dublin, Ireland) (Larkin et al., 2007). The *Alu* element is named after its recognition site for the restriction endonuclease *Alu*I (5′-AGCT-3′) and belongs to the short-interspersed elements (SINEs) family (Hancks and Kazazian, 2012). *Alu* elements are generally 300 bp long and have more than 500 thousand copies in the human genome, constituting ~10% of all nucleotide sequences (Deininger and Batzer, 1999; Babatz and Burns, 2013; Kaer and Speek, 2013). *Alu* elements share a high level of sequence identity and consist of three major lineages classified by the 18 diagnostic nucleotides in them (Shen et al., 1991; Kim et al., 2016): *Alu*J, *Alu*S, and *Alu*Y. The *Alu* element is a type of mobile element called retrotransposons, which amplify through a copy-and-paste mechanism (Callinan and Batzer, 2006). The *Alu* element can alter the human genome by either a *de novo* insertion event or *Alu*-mediated genomic rearrangement, resulting in a disruption of the open reading frame (ORF) via direct insertion into the exons, alternative splicing, genetic deletions, and duplications or conversions by homologous recombination (HR) between *Alu* elements. *Alu*-mediated genomic rearrangements are present in two forms: non-homologous end joining (NHEJ) and non-allelic homologous recombination (NAHR), which is a special form of HR, and the latter can be subdivided into interchromosomal and intrachromosomal NAHR (Kim et al., 2016). There have been estimations that mutagenesis associated with *Alu* elements may account for 0.1–0.3% of human genetic disorders (Callinan and Batzer, 2006; Kim et al., 2016; Geng et al., 2018). Hence, we hypothesized that the intragenic deletion in which either side of the breakpoints was located within two *Alu* elements with high sequence identity occurring via the intrachromosomal recombination mechanism where the two repetitive sequences crossed over within the same gene. The identical 13-bp segment might act as the core sequence in the *Alu* recombination-mediated deletion (Figure 3).

To investigate how the intragenic deletion containing exon 10 affected the expression mode of *MFSD8*, cDNA analysis through RT-PCR and T-clone sequencing was performed for individuals both with (G065-1 and G065-3) and without (G065-2) the mutation (Figure 4). Multiple sequences that lacked one or two of the alternatively spliced exons 6–8 (absence of exons 6, 7, 6 and 7, or 7 and 8) were detected in the RNA of peripheral blood collected from all participants. The loss of exon 10 was identified in the heterozygotes of the gross deletion in conformity with our expectations. However, the absence of exon 11 was ubiquitously observed in various transcripts regardless of the carrier status of the deletion or repetition of the experiments and was the predominant form for most transcripts detected except for the common whole-length transcript, like NM_152778.4. Since exon 11 is present in all 10 currently known transcripts in the UCSC Genome Browser database, it was substantially unlikely that the loss of exon 11 originated from alternative splicing. Further research into the expression pattern of the gene *MFSD8* might be needed given that only the samples of peripheral blood were analyzed in this study.

Despite the negative results of CNV-seq and biomedical screening analysis for most probands in this study, pathogenic CNVs and IEM are responsible for a considerable number of epilepsy cases. Multiple research studies have suggested that P/LP CNVs have a relatively high detection rate (10.7–16.1%) in epilepsy patients with other comorbidities or negative for pathogenic SNVs (Borlot et al., 2017; Tsuchida et al., 2018; Coppola et al., 2019), compared to <3% detected in those with common epilepsy (Niestroj et al., 2020). In addition, a certain number of IEM could contribute to metabolic dysfunctions in biomedical pathways targeting neuronal networks and manifest epileptic seizures and other neurological conditions that are responsible for a large portion of monogenic disorders involved with epileptic seizures (Vitiello et al., 2012; Sharma and Prasad, 2017; Tumiene et al., 2018; Reddy and Saini, 2021). Thus, it is essential to perform a comprehensive molecular examination consisting of WES, CNV-seq, and biomedical analysis (like MS/MS and GC-MS) for clinically undiagnosed patients with epilepsy and other neurological comorbidities.

In conclusion, we made the molecular diagnosis in seven unrelated Chinese families with genetic epilepsy. Eleven causative monogenic variants were identified in seven genes responsible for the corresponding disorders in the pedigrees, including six novel variants (c.1408T>G in *ALDH7A1*, c.1994_1997del in *CDKL5*, c.794G>A in *QARS1*, c.2453C>T in *GRIN2A*, and c.217dup and c.863+995_998+1480del in *MFSD8*). To the best of our knowledge, this is the first study to associate the gross deletion in *MFSD8* with the mechanism of *Alu*-mediated genomic rearrangements, which might shed light on the pathogenesis of this genetic disorder. The findings of this study were expected to provide guidance to the treatment, prognosis, genetic counseling, and prenatal diagnosis for these families and new insights into the pathogenic mechanism of genetic epilepsy.

## Data availability statement

The original contributions presented in the study are included in the article/Supplementary material, further inquiries can be directed to the corresponding authors.

## Ethics statement

The studies involving human participants were reviewed and approved by Institutional Review Board of Fujian Maternity and Child Health Hospital (2023KYLLR01016). Written informed consent to participate in this study was provided by the participants' legal guardian/next of kin.

## Author contributions

BM: writing—original draft preparation, investigation, and formal analysis. NL, DG, DH, and HX: resources and data curation. LC, QH, and MZ: resources. MC: validation. HH: writing—review and editing and supervision. LX: supervision. All authors contributed to the article and approved the submitted version.
