# Supplementary material for: Molecular analysis and prenatal diagnosis of seven Chinese families with genetic epilepsy
Source: Front Neurosci. 2023 May 12;17:1165601. doi: 10.3389/fnins.2023.1165601 (PMC10213446; doi:10.3389/fnins.2023.1165601)
Supplement: Supplementary file 1 [file Data_Sheet_1.PDF]

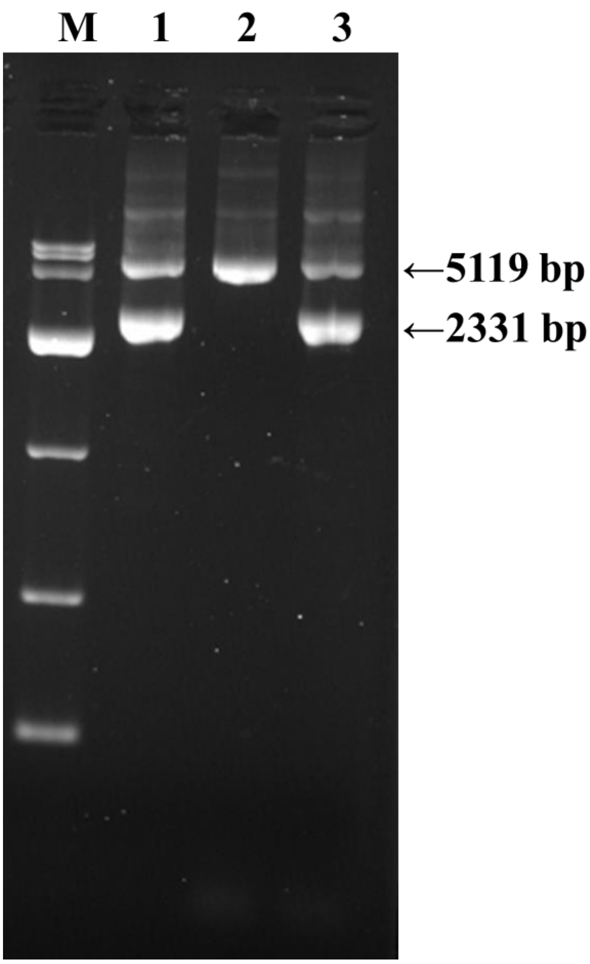

Figure S1 The gap-PCR suggested a gross deletion in family G065.

Primer pairs of *MFSD8*-Gap-F and *MFSD8*-Gap2R (Table S1) were used to amplify the fragments covering the 2788-bp deletion. The results indicated that the proband G065-1 and her mother G065-3 were heterozygous carriers of the intragenic deletion. M, DL 10000 DNA Marker; 1, G065-1; 2, G065-2; 3, G065-3.
